# Hatching on-farm under field conditions –a comparison of fear-related response and urofecal glucocorticoid metabolite levels in broiler chicks hatched on-farm and in the hatchery

**DOI:** 10.1016/j.psj.2026.107358

**Published:** 2026-06-27

**Authors:** Sabine Christa Margarete Vossler, Anna Schwarz, Stephanie Schäfers, Chadi Touma, Tanja Wolf, Nicole Kemper, Birgit Spindler

**Affiliations:** a Institute of Animal Hygiene, Animal Welfare and Farm Animal Behaviour (ITTN), University of Veterinary Medicine Hannover, Foundation, 30173, Hannover, Germany; b Department of Behavioural Biology, Osnabrück University, 49076, Osnabrück, Germany

**Keywords:** On-farm hatching, Broiler, Glucocorticoid metabolite, Behavior test

## Abstract

Broiler chicks are usually hatched in hatcheries, sorted, and transported to farms, which can cause stress. Alternatively, transporting eggs to farms and hatching chicks in their future environment may reduce stress and affect their behavior.

The study was conducted on conventional farms to evaluate whether on-farm hatching can improve animal welfare. Behavioral responses to humans and novel objects, as well as stress levels measured by urofecal glucocorticoid metabolites (ufGCM) were compared between on-farm hatched (OH) and hatchery-hatched (HH) broiler chickens. Slow-growing broiler chickens were monitored on 1 farm comprising 2 houses, each containing approximately 5,700 chickens. Production parameters were recorded over 7 production periods (PP). The Human Approach Test (HAT) and Novel Object Test (NOT) were performed on day of life (DL) 1 and 7 and latency parameters were assessed (n = 7 PP). Fecal samples for ufGCM analysis were collected at DL 0, 1, 2, 4, and 5 (n = 2 PP).

More OH chicks were counted in HAT on both observation days than in HH. A significant difference in latency to enter the 1 m radius was observed between DL 1 and 7 (*P* = 0.015). For NOT, significant latency differences appeared between hatching groups (25 cm radius: *P* = 0.004; touch: *P* < 0.001) and for touch latency between DL (*P* < 0.001). HH chicks showed slightly higher ufGCM values than OH chicks. Both groups started with a high ufGCM level on DL 1 (OH = 1,526.73 ng/g; SD = 439.63 ng/g; HH = 1,730.72 ng/g; SD = 399.84 ng/g), which dropped to approximately 400 ng/g at DL 4 and DL 5. The only difference in the production parameters was found in live weight at DL 1, 7, and 42 (OH > HH, *P* < 0.001).

This field study showed that chicks hatched on-farm were more accustomed to humans, less fearful, and exhibited slightly lower stress hormone levels than hatchery-hatched chicks. However, a larger sample is needed to confirm these findings.

## Introduction

Broiler meat production accounts for up to 80% of poultry meat production and has steadily grown worldwide over the years (BMEL, 2024). Broiler chickens usually hatch in specialized hatcheries at day 21 of incubation. The hatching window spans 12 to 24 hours, during which most chicks hatch (Mather et al., 1976). The individual hatching process of one chick can last from 4 to 24 hours and affects, for example, later-life weight (Løtvedt et al., 2014). After hatching, non-viable or malformed chicks are sorted out in the hatchery, and the remaining chicks are vaccinated before they are loaded into crates for transportation to the rearing house under climate-controlled conditions. The European Nations allows the transport of chicks without access to food or water for up to 24 hours (EU Council Directive, 2005) as does national law provided they arrived at the farm within 60 hours of hatching (TierSchTrV, 2009). The yolk sac is the only source of nutrients for the newly hatched chick until it has access to food (Romanoff, 1960). It was assumed that this provides the chick with nutrients for the first 72 hours (Freeman et al., 1974). It is now assumed that the yolk sac does not provide the necessary amount of nutrients to fully promote the genetic expression of the growth potential of today's common broiler genetics and the development of the gastrointestinal tract and immune system (Panda et al., 2015). Additionally, due to transport and handling in the hatchery, the chicks are exposed to stress factors such as rough handling, sex-sorting, and no access to food (Elfwing et al., 2015; Ericsson et al., 2016; Hedlund et al., 2019; Molenaar et al., 2023).

An alternative to hatchery hatching is on-farm hatching. The hatching eggs are transported to the poultry house at day 18 of incubation (at this time point, the eggs in the hatchery are transferred from the incubator to the hatching cabinet), where the chicks then hatch in their future living environment. There is no need to handle or transport the chicks, and they have immediate access to food and water. On-farm hatching is increasingly used in European countries, such as the Netherlands (Innovate Animal AG, 2023). In Germany this hatching routine is not yet common. Nevertheless, during avian influenza outbreaks, when restriction zones must be established, and chick transport is prohibited (Geflügelpest-Verordnung, 2018), on-farm hatching is becoming increasingly common, as the transport of hatching eggs is possible upon request (EU Regulation, 2020).

On-farm hatching is therefore expected to have a positive effect on animal health, performance, and welfare (de Jong et al., 2019) and to reduce stress (Hedlund et al., 2019) because the chicks are not sorted and transported without access to feed. Until now, the influence of the on-farm hatching on life weight (Løtvedt et al., 2014; Souza da Silva et al., 2021) and behavior (Giersberg et al., 2020) of the chickens has only been investigated in some studies under experimental conditions. De Jong et al. (2020) found that in small experimental units using on-farm hatching, the weights of on-farm hatched chicks were significantly higher and mortality rates were significantly lower.

To record and evaluate animal behavior in poultry farming, fear-related tests are used for behavioral observation. The Human Approach Test (**HAT**) examines the animal's voluntary approach to a non-moving human. This test form is particularly suitable for poultry in cage-free housing, is quick and easy to perform, and provides good information about the relationship between the tested animals and humans (Graml et al., 2008). The Novel Object Test (**NOT**) is a method for determining general anxiety and neophobia. It is considered fast and practicable even in large flocks (Fraisse et al., 2006; Forkman et al., 2007). These so-called fear tests are considered well established in laying hens. So far, however, only a few studies have investigated anxiety in broilers. Under experimental conditions, Giersberg et al. (2021) found that in these tests, all responses measured in the behavioral tests were affected by age; in later life, chickens were significantly less fearful than during the first days of life (**DL**). The hatching system had no influence.

Whether hatching on-farm affects the stress levels of broiler chicks compared to hatchery hatching has not yet been objectively investigated. The adrenocortical hormone corticosterone is suitable for assessing animal welfare aspects of stress (Möstl et al., 2002). In general, corticosterone can be measured in poultry blood (Afrouziyeh et al., 2022) or feces (Rettenbacher et al., 2004).

Corticosterone is produced in the adrenal cortex and its release is regulated by the hypothalamus-pituitary-adrenal axis (Axelrod et al., 1984). It circulates in the blood, is metabolized by the liver, and is excreted into the gut via the bile duct or into the urine via the kidneys. Corticosterone metabolites can be measured a few hours after the stressor acts on the animal and are less affected by diurnal secretion (Touma et al., 2005).

In chicken, the time delay between the stress event and detection in the feces is between 1.5 hours (1st peak) and 4.5 hours (2nd peak); the first peak results from renal excretion and the second peak is caused by elimination via the gut (Rettenbacher et al., 2004). It must be noted that corticosterone degradation occurs in the feces. Therefore, an analysis of freshly deposited feces or up to 4 hours afterwards is necessary; otherwise, if the time interval is extended, degradation processes will take place (Wolf et al., 2024).

As corticosterone circulates in the blood, the simplest method is to measure its concentration by taking a blood sample. However, this is associated with considerable stress due to the need to catch and restrain the animals. Depending on the duration of the blood sampling procedure, this can lead to falsely reported concentrations (Romero et al., 2005).

To evaluate whether on-farm hatching can improve animal welfare by reducing stress levels and influencing fear-related behavior in chicks, this field study aimed to compare behavior in specific fear-related tests and corticosterone metabolite levels in the feces of on-farm-hatched and hatchery-hatched chicks.

## Material and methods

### Ethical statement

All of the animals were housed in accordance with European Union (EU) (EU Council-Directive, 2007) and German national law (TierSchG, 2022; TierSchNutzV, 2021). In compliance with European Directive 2010/63/EU Article 1, 5. (f), the present study used non-invasive procedures. Therefore, the study was approved by the research ethics committee of the University of Veterinary Medicine Hannover, Hannover, Germany (TVG-2025-V-7).

### Animals and housing conditions

This field study was conducted on a farm with 2 identical forced-ventilated broiler houses (470,64m², each) in Lower Saxony, Germany.

During our study, the farm participated in the entry level (1 gold star) of the animal welfare label of the German Animal Welfare Federation (German Animal Welfare Federation, 2017), with stocking density up to 29 kg/m² at slaughter, access to an outdoor scratching area, in this case a winter garden (from DL 21), as well as enrichment material (straw bales, pecking stones and elevated platforms) from day one, with at least two items per 150 m^2^. This label also requires the fattening of slow-growing genetics. Up to 6,400 Ranger Classic broilers (Aviagen Inc., Huntsville, AL, USA) were kept in each house for 42-45 fattening days, with a calculated live weight of 2,196 g (Aviagen, 2018). On-farm hatched (**OH**) and hatchery hatched (**HH**) chicks were fattened simultaneously in 2 separate houses and came from the same parent flock within each production period (**PP**). The eggs of both groups were incubated up to 18 incubation days in the hatchery (BWE-Brüterei Weser-Ems GmbH & Co. KG, Rechterfeld, Germany). At day 18 of incubation (or DL −3), the eggs of the OH group were transported in air-conditiond trucks (about 2 hours) to the poultry house about 3 days before hatching. The eggs of the HH group were kept in the hatchery and hatched in the hatcher cabinet. In total, 7 production periods were investigated from July 2023 to September 2024. The production week (**PW**) of the different parental stock ranged from 17 to 38 weeks across different production periods (Table 2. After each production period, the houses for the OH and HH chicks were swapped to rule out any housing-related effects in the analysis.

Both houses were littered with approx. 800 – 1,000 g/m² wood shavings and heated at 33-35°C air temperature before housing HH or OH chicks. Air humidity was between 40% and 50%. The light program was set up to 18 h light and 6 h darkness until the end of the PP. Food and water were provided ad libitum. The chicks were fed a commercial fattening diet following a 4-phase feeding scheme: starter, grower 1 and 2, and finisher.

Each house was equipped with 3 automated feeder pan lines and 4 nipple drinker lines. Feed was also offered on 3 lines of chick paper for the first days of life, with around 40 g starter feed/chick and around 30 g/chick in the feeding troughs. A feed conversation rate could not be calculated because both houses had only one feed silo and the feed line was connected in series.

The mean temperature in the house for on-farm hatching was at DL −2 up to DL 0 33.88°C (SD 0.47°C) and the air humidity 39.56% (SD 2.20%) (Table 1). At DL 1, the mean air temperature and humidity over all 7 PP and both houses were 32.7°C and 57%, respectively. At DL 2, the mean was 32.34°C and 60%, at DL 4, 31.15°C and 59%, and 30.36°C and 61% at DL 5. At DL 7, the mean air temperature and humidity were 29.17°C and 63% and at DL 42 21.65°C and 79%, respectively for both hatching groups (for more details see Supplementary Table A).

**Table 1:** Mean temperature and air humidity in the barn for on-farm hatched chicks (OH) from day of life (DL) −2 up to 0 (day 18 to 21 of incubation) and hatchery hatched chicks (HH) at DL 0.

Hatch- form		Day of Life	Temperature (°C)	Relative air humidity (%)
	Mean	−2	34.13 (SD 0.34)	37.17 (SD 9.05)
OH	Mean	−1	34.17 (SD 0.45)	39.99 (SD 9.78)
	Mean	0	33.34 (SD 0.64)	41.51 (SD 9.08)
HH	Mean	0	34.09 (SD 0.65)	54.71 (SD 6.18)

### On-farm hatching (OH)

At day 18 of incubation, a varying number of hatching eggs were laid in one house. The plastic transportation trays (73 eggs per tray) were placed 10-15 cm next to the chick paper with feed, and as close as 20 cm to the nearest water line. For better insulation, the trays were placed on corrugated cardboard sheets. During the period from day 18 to 21 of incubation (DL −3 to DL 0), the eggshell temperature was monitored using 6 eggshell sensors (Mach-Sens BV., Lichtenvoorde, The Netherlands). From the time the eggs were placed in the house (DL −3) until DL 0, continuous lighting was maintained in the OH barn to ensure the chicks could find food and water immediately after hatching. On DL 0, the lights were on continuously in both barns. On Day 1, a 4-hour period of darkness was scheduled. The lights were on from 3:00 a.m. to 11:00 p.m. Starting on DL 2, there was an 8-hour dark period. The lights were on from 5:00 a.m. to 9:00 p.m. In addition to artificial lighting, daylight entered through windows on the long sides of the barns.

After hatching at DL 0, the non-viable chicks were selected using the same standards as in the hatchery, and the eggshells were removed from the house.

At DL 1, the chicks were vaccinated against infectious bronchitis via spray in the poultry house.

### Hatchery hatched (HH)

The chicks from the hatchery were hatched at day 21 of incubation (DL 0) in the hatcher cabinet (in the dark). After hatching, they underwent quality control, during which non-viable chicks were sorted out and fit chicks placed in transport baskets (100 chicks/basket). In the baskets, they get a spray vaccination against infectious bronchitis. The transportation time from the hatchery to the farm was about 2 hours. In the poultry house (DL 0), the chicks were placed on the chick paper for direct access to feed. The climate conditions at DL 0 when the hatchery chicks arrived in the house are presented in Table 1.

### Data collection - performance data

Production data, including hatchability and mortality, were documented for each hatching group across the 7 PP (Table 2).

**Table 2:** Production data from on-farm hatched (OH) and hatchery hatched (HH) chicken flocks (Genetic Ranger Classic, n = 7 production periods (PP) per hatching group); DL = Day of life; PW = Production week parent stock; *DL 35; significant differences *P* < 0.05;**/** Insufficient data, calculation impossible.

Production period (PP)	Hatch- form	PW parent stock	Number of chicks DL 0	Hatch-ability (%)	Pasga score	7-day mortality (%)	Mortality in total (%)	Live weight DL 1 (g)	Live weight DL 7 (g)	Live weight DL 42 (g) (* DL 35)
PP1	OH	22	6,113	88.15	9.56	0.67	2.08	57.88	191.99	2,428.98
	HH	22	5,800	88.00	9.58	1.09	2.93	52.04	178.02	2,320.16
PP2	OH	31	5,537	84.28	9.5	1.34	2.89	59.76	191.86	2,497.35
	HH	31	5,700	84.24	9.66	1.14	2.28	52.56	173.36	2,552.69
PP3	OH	38	5,004	85.75	9.44	4.3	6.49	57.3	187.03	2,406.72
	HH	38	5,700	79.18	9.52	2.82	4.67	55.74	182.76	2,452.05
PP4	OH	18	6,356	89.76	9.6	1.13	3.15	48.52	164.45	2,308.29
	HH	18	5,900	85.99	9.78	0.93	3.14	50.68	169.12	2,427.27
PP5	OH	27	5,722	86.37	9.96	1.43	2.78	52.02	177.6	2,427.03
	HH	27	5,800	87.74	10	1.41	3.07	50.95	171.47	2,207.33
PP6	OH	17	5,499	87.59	9.68	0.87	2.2	52.38	122.71	*1,848.09
	HH	17	5,500	86.70	9.84	1.75	3.56	49.92	107.26	*1,807.72
PP7	OH	18	5,788	89.09	9.8	0.9	2.82	52.94	147.94	2,312.09
	HH	18	5,600	90.00	9.82	1.86	3.52	48.78	132.44	2,276.7
										
Mean	OH	/	5,717	87.28	9.65	1.52	3.20	54.40	169.08	2,318.36
SD	OH	/	439.1	1.93	0.18	1.26	1.50	3.99	26.00	218.02
Mean	HH	/	5,714	85.98	9.74	1.57	3.31	51.52	159.20	2,291.99
SD	HH	/	134.5	3.49	0.17	0.65	0.74	2.25	28.20	243.10
P-value	Hatch-form	/	/	/	0.079	0.925	0.209	< 0.001	<0.001	< 0.001
	Hatch-form *House	/	/	/	0.667	/	/	0.627	0.475	0.435
	Hatch-form *PP	/	/	/	< 0.001	/	/	< 0.001	< 0.001	/

The hatchability is usually calculated from the number of eggs that are placed in the incubator minus the unfertilised eggs and late dead chicks that are sorted out during the handling of the eggs (at day 7 and 18 of incubation), and the number of hatched chicks that are delivered to the farm after the quality control and the selection of the non-viable chicks. In this on-farm study, technical issues prevented sorting late-dead embryos in the hatchery; they were also delivered to the poultry house. Therefore, the hatching rates here are calculated from the varying number of eggs delivered to the barn, less the unhatched eggs (including unfertilized eggs and eggs from which no chick hatched), and the chicks selected at DL 0.

At DL 1, DL 7, and DL 42, a total of 100 randomly selected chicks per flock were weighed, except for the PP 6, where the weights of DL 35 were available instead of DL 42 (manual poultry scales BAT1, VEIT Electronics, s.r.o., Moravany, Czech Republic). The average chick quality was determined from 50 chicks per flock, using the Pasgar©Score (Boerjan, 2002) at DL 1. Therefore, the characteristics: navel, beak, legs, belly, and reflexes were assessed. Chicks without deviation from the norm received 10 points. For each deviation from the norm, 1 point was deducted for one of the characteristics, so that a minimum of 5 and a maximum of 10 points could be awarded.

### Behavioral tests

In this study, 2 behavioral observation tests, according to the Welfare Quality® (Welfare Quality, 2009) Assessment Protocol for Poultry, modified by Giersberg et al. (2021), were conducted at DL 1 and DL 7 over 7 PP in both hatching groups (OH and HH). For this, the Human Approach (HAT) and the Novel Object Test (NOT) were used. HAT and NOT were performed on both days at 8 locations per house, evenly spaced out throughout the entire barn. In the HAT, the number of chicks within a 1-meter radius of a squatting person was recorded every 30 seconds for 3 minutes. During the Novel Object Test (NOT), conducted immediately after the Human Approach Test in the same locations within the broiler house, the animals were presented with 2 unfamiliar three-dimensional objects—a pink textmaker (DL 1) and a colored pole (DL 7)—which they could freely approach and inspect for 3 minutes.

The number of animals within a 25 cm radius of the object was recorded every 30 seconds for a total of 3 minutes. In both tests, the latency time to the first animal entering the radius or to the first animal touching the object/human was also recorded. If no animal entered the radius or touched the object/ human, the maximum examination time of 180 sec was noted. One person served as the observer throughout the study and was aware of the hatching groups.

### Fecal samples

Fecal samples for analysis of urofecal glucocorticoid metabolites (ufGCM) were collected over 2 production periods (PP 6 and PP 7) in both hatching groups (HH and OH).

To collect fecal samples, 3 plastic sheets (1 m x 1 m) were laid out at various places in each broiler house, and all droppings were collected from these sheets approximately 4 hours later. New plastic sheets were then laid out to ensure the collected samples were no older than 4 hours. The droppings on each sheet were collected separately and mixed into 3 independent pool samples, so that 3 measured values were available at each collection time. The first urofecal sample collection took place at DL 0, the day on which the day-old chicks were transported to the farm, where they arrived around noon. A total of 4 and 3 collections were made in both houses (OH and HH), respectively, throughout the day, at 10:00 (only OH), 13:00, 16:00, and 19:00. On DL 1, again 4 collections were made at the same times for both hatching groups. 3 more collections were made at DL 2 (13:00, 16:00, 19:00), and at DL 4 and 5, 1 collection was made on both mornings (10:00). This resulted in a total of 78 samples across 2 PP for OH and 72 for HH. Each pooled sample had an approximate wet weight of 2.5 g, which corresponds to about 3-5 individual urofecal samples. The samples were frozen at −20°C immediately after collection and later freeze-dried (Freeze-Drying System Alpha 1-4, Martin Christ Gefriertrockungsanlagen GmbH, Osterode am Harz, Germany). After that, the sample's dry weight was about 0.5 g. The analysis took place at the University of Osnabrück, Germany (Department of Behavioral Biology) using a validated enzyme-immunoassay and established protocols to quantify ufGCM concentrations in chicken droppings (Rettenbacher et al., 2004; Wolf et al., 2024). According to the observations, the chicks did not notice the plastic sheet, as they neither avoided nor approached it more frequently. Therefore, a bias toward confident chicks is unlikely.

### Statistical analysis

All data were recorded using Microsoft Excel (version 2016; Microsoft Corporation, Redmond,WA, USA) and processed descriptively (including mean value, minimum, maximum, standard deviation (SD)).

Statistical analysis was conducted using R 4.5.0 (Integrated Development for R. RStudio, PBC, Boston, MA, USA, 2025).

Descriptive statistics, calculating mean values, and standard deviations were used on all assessed parameters. The data and residuals were checked for normal distribution using Q-Q-plots and Shapiro Wilk tests. All parameters and residuals except mortalities and ufGCM were distributed normally.

Normally distributed parameters were analyzed with linear mixed models using the lme4 procedure with the hatching method as a fixed factor, and the house and production period as random factors as well as the interactions between these factors (Bates et al., 2015).

The Mann-Whitney U test was used to evaluate the influence of the hatching method on 7-day mortality and total mortality (R Core Team, 2025).

For analysis of ufGCM, the homogeneity of variances was tested using Levene's test from the car-package of R (Fox J, Weisberg S, 2019; _An R Companion to Applied Regression_). To adjust for the right-skewed heteroskedasticity, a generalized linear model (GLM) using a Gamma distribution with a logarithmic link function was modeled with hatching method and day of fattening and their interaction. Each parameter was analyzed separately.

*P* < 0.05 was regarded as significant, while 0.10 < *P* < 0.05 was seen as a tendency.

## Results

### Production data

Table 2 shows the production data for each production period and hatching group. The mean hatchability achieved on-farm was comparable to the one of HH.

The average live weight at DL 1 for the HH group was in accordance with the Ranger Classic performance guide (Aviagen, 2018), and the OH chicks were significantly heavier at DL 1 (*P* < 0.001) and DL 7 (*P* < 0.001). This difference was even higher at the end of fattening (DL 42), when the OH chicks had a 26 g higher weight than HH chicks (*P* < 0.001). There was an interaction between weighing days and the different PP within a hatching group (*P* < 0.001), but not between barns.

There were no significant differences between the 2 hatching forms in terms of 7-day mortality (*P* = 0.925) and total mortality (42 fattening days; *P* = 0.209). A trend toward a difference in the mean Pasgar^ࣩ^ Score across all 7 PP is observable (*P* = 0.079).

### Behavioral tests - number of chicks

**Fig. 1:**
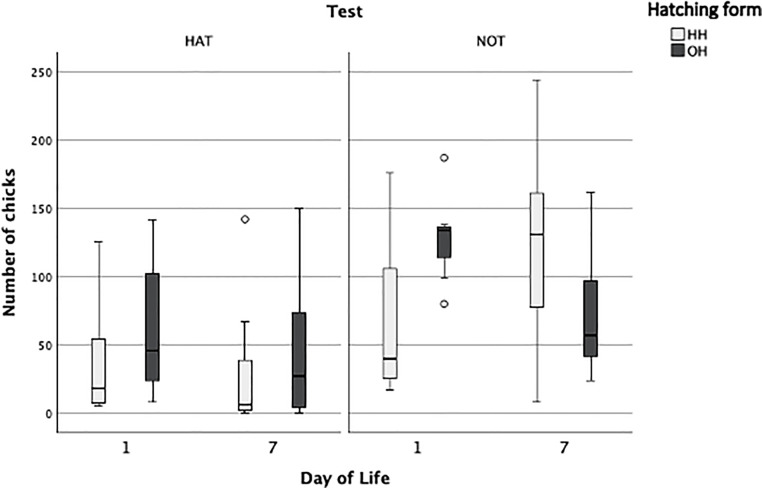
Median number of broiler chicks (Ranger classic) approaching at Day of Life (DL) 1 and DL 7 in the Human Approach Test (HAT) and the Novel Object Test (NOT). Each box represents the 1st and 3rd quartile (upper and lower line) as well as the median (middle line); the whiskers represent the minimum and maximum; outliers are represented by the circels (t-test, not significant) (n = 7 production periods and 8 locations per hatching form and day).

During the Human Approach Test at DL 1, the median number of OH chicks entering the 1 m radius was with a median of 46 chicks almost 2.5 times higher than that of HH chicks (median: 18 chicks). By DL 7 in median 27 OH and 6 HH chicks were counted (Fig. 1).

In the Novel Object Test, 3 times more OH chicks than HH chicks (134 OH chicks vs. 40 HH chicks) entered the radius at DL 1 (*P* = 0.362). By DL 7, only half as many OH chicks entered as HH chicks (median: OH: 61 vs. HH 131, (Fig. 1)). There was a significant difference in chick numbers between the HAT and NOT (*P* < 0.001), but not between hatch-forms (*P* = 0.244; see Table 3).

**Table 3:** Statistical Analysis of the Behavioral Tests, Human Approach Test (HAT) and Novel Object Test (NOT) using R 4.5.0 (Integrated Development for R. RStudio, PBC, Boston, MA, USA),with a t-test, fixed factor: hatching method, significant *p* < 0.05; Day of life = DL, Hatching form = HF.

	P-values				
Parameter	Numbers of chicks	HAT latency 1 m	HAT latency touch	NOT latency 25 cm	NOT latency touch
Test	< 0.001	-	-	-	-
DL	0.914	0.015	0.964	0.818	< 0.001
Hatching form	0.244	0.297	0.121	0.004	< 0.001
DL*HF	0.226	-	-	-	-
DL1*HF	-	0.134	0.254	< 0.001	< 0.001
DL7*HF	-	0.979	0.287	0.459	0.335
Test*DL	0.451	-	-	-	-
Test*HF	0.979	-	-	-	-
Test*DL*HF	0.362	-	-	-	-

### Latency

The latency in the HAT differed significantly between the DL for entering the 1 m radius (*P* = 0.015), but not between the hatching forms in the period before the radius was entered (*P* = 0.297), or until the human was touched (*P* = 0.121) (Table 3, Table 4).

**Table 4:** Measured times (in seconds; s) for the latency in the Human Approach Test (HAT) with broiler chicks (Ranger classic) per hatching form (OH = On-farm hatching; HH = Hatchery-hatched) and DL = Day of Life on entering the 1 m radius and touching the human; Max observation time of 180 s, was noted when no chick entered the radius or touched the human (n = 7 production periods and 8 locations per hatching form and day).

DL	Hatching form	Latency HAT	Min (s)	Max (s)	Mean value (s)	Median (s)	SD (s)
1	OH	Radius 1m	1	180	96.2	95	77.98
	HH	Radius 1m	1	180	117.46	180	76.31
	OH	Touch	180	180	180	180	0.00
	HH	Touch	3	180	176.84	180	23.65
7	OH	Radius 1m	1	180	131.59	180	70.91
	HH	Radius 1m	1	180	131.21	180	73.71
	OH	Touch	180	180	180	180	0.00
	HH	Touch	53	180	177.02	180	17.70

**Fig. 2:**
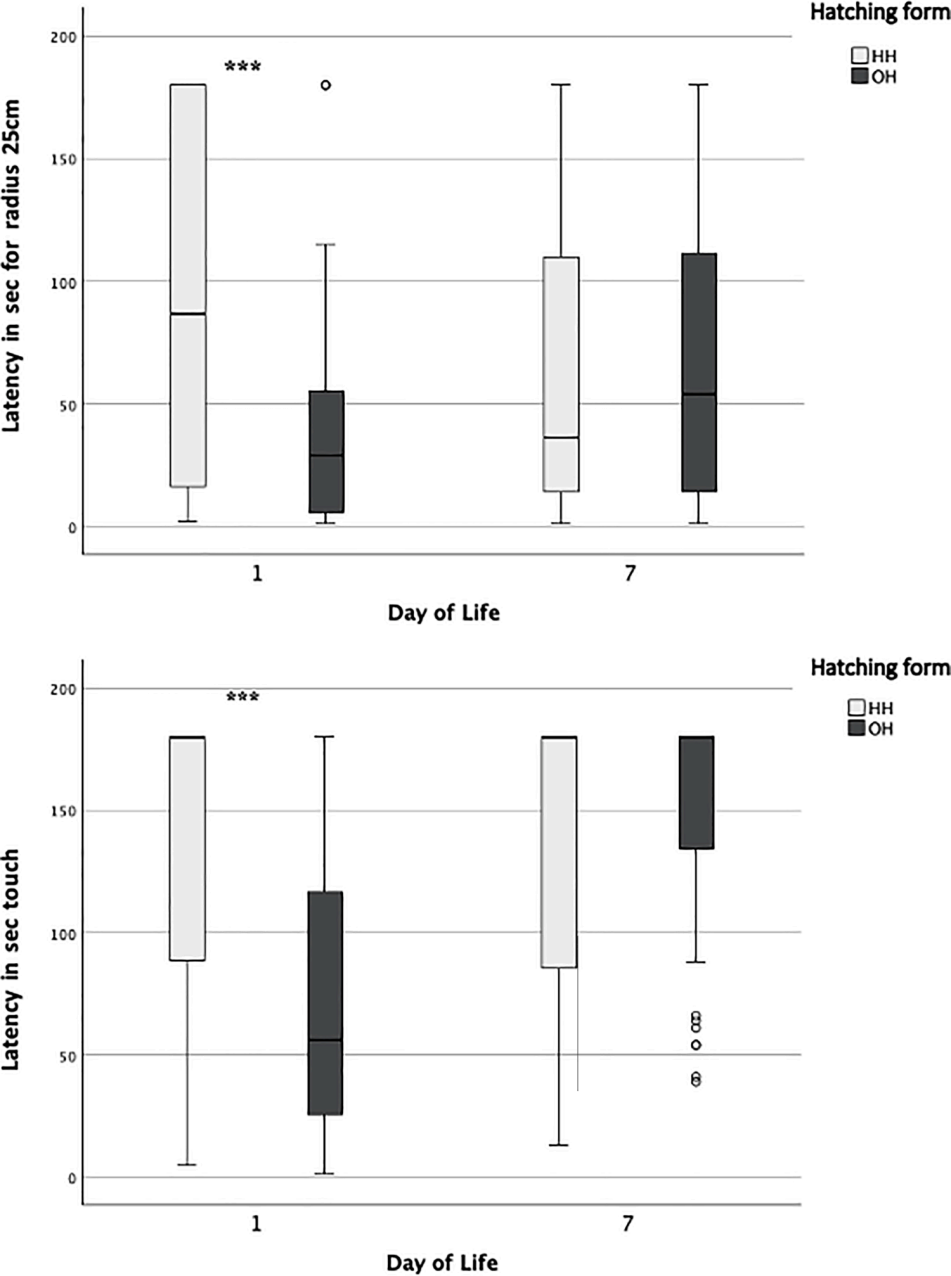
a/b Latency in the novel object test (NOT) of the broiler chicks (Ranger classic) for entering the 25 cm radius (a) and touching (b) the novel object in seconds at Days of Life 1 and 7 for on-farm hatched (OH) and hatchery-hatched (HH) chickens. The maximum observation time was 180 sec and was noted when non-interaction with the novel object, no entering or touching was observed. Each box represents the 1st and 3rd quartile (upper and lower line) as well as the median (middle line); the whiskers represent the minimum and maximum; outliers are represented by circels (t-test; *** = *P* < 0.001).

The latency in the NOT differed significantly between OH and HH entering the 25 cm radius as well as touching the object, where the HH chicks took twice as long as the OH chicks (both *P* < 0.001) at DL 1 (Fig. 2).

At DL 7 there were no significant differences between the hatching forms in the time until the radius was entered (*P* = 0.459) or until the human was touched (*P* = 0.335).

For detailed descriptive statistics, see Supplementary Tables B-C.

### Urofecal corticosterone metabolites

The concentrations of urofecal corticosterone metabolites (ufGCM) revealed a strong tendency towards a difference (*P* = 0.057) between the two hatching forms (Table 5). With an overall mean of 1,006.74 ng/g (SD 543.27 ng/g), the ufGCM concentration was slightly lower in the OH group compared to the HH group (mean: 1,019.82 ng/g, SD 491.36 ng/g).

**Table 5:** Statistical analysis of the urofecal glucocorticoid metabolites (ufGCM) concentrations using R 4.5.0 (Integrated Development for R. RStudio, PBC, Boston, MA, USA),with a generalized linear model (GLM), fixed factor: hatching form, significant *P* < 0.05; 0.10 < *P* < 0.05 was regarded as a tendency; Day of life (DL), Hatching form (HF).

	P-values
Parameter	ufGCM
Hatching form	0.057
Day of life	< 0.001
HF*DL	0.621

**Fig. 3:**
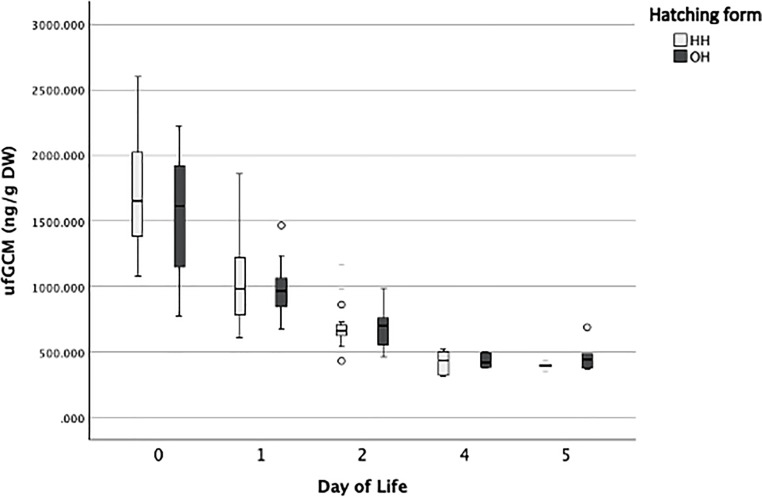
Urofecal glucocorticoid metabolite (ufGCM) concentrations of broiler chicks (Ranger Classic) of both hatching forms ((OH: on-farm hatched; HH: hatchery-hatched) over days of life 0, 1, 2, 4, 5 (n = 78 OH and n = 72 HH urofecal samples); each box represents the 1st and 3rd quartile (upper and lower line) as well as the median (middle line); the whiskers represent the minimum and maximum; outliers are represented by the circles (all t-tests, not significant).

Fig. 3 shows the ufGCM concentration of the broiler chicks across the first 5 days of life. There was no significant interaction effect of DL and hatching form on the ufGCM concentration (*P* = 0.621).

In general, the mean ufGCM content was highest at DL 0 for both hatching forms, decreasing thereafter to a basal value around 400 ng/g from fattening day 4 onward.

Nevertheless at DL 0, the HH chicks showed a slightly higher ufGCM content, with a mean value of 1,730.73 ng/g (SD 399.84 ng/g) than the OH chicks with 1,562.73 ng/g (SD 439.63 ng/g). In both hatching forms, the concentration already decreased on the following days of life. At DL 4 and DL 5, it seemed that the ufGCM concentrations reached a stable value, and the SDs were lower than on the first days after hatching.

## Discussion

This on-farm study aimed to compare the behavior and stress levels of hatchery chicks with those of chicks hatched on a conventional broiler fattening farm. To investigate whether hatching in the barn leads to greater animal welfare due to lower stress levels among the animals, fear-related tests were performed and fecal samples for corticosterone metabolites were collected to assess stress levels. Additionally, production parameters were compared between the two hatching forms.

### Performance data

Under the climatic conditions of this experiment, on-farm hatching was effective, with a hatching rate nearly identical to that observed in the hatchery. Direct comparison of hatching rates or the number of chicks in the barn on DL 0 is challenging because unsorted eggs were delivered to the house. For each PP, the hatchery delivered a variable number of eggs. The quantity was determined using the projected hatch rate and the presumed number of eggs containing viable chicks.

Live weight was significantly higher on all sampling days for the on-farm hatched (OH) chicks compared to those from the hatchery. The OH chicks had immediate access to feed and water after hatching. In this experiment, the hatchery-hatched (HH) chicks arrived at the barn approximately 27:55 h after the first on-farm chicks had hatched, as Hofmann et al. (2025) observed in 2 PP. As both groups originated from the same parent flock and were stored under identical conditions until day 18 of incubation, it is likely that the hatch window was similar for both methods. The observed interactions in weights and Pasgar^ࣩ^ Score (*P* < 0.001) within one hatching group among individual PP may be likely due to the use of 4 different parent flocks with varying PW for 7 PP, which precludes direct comparison.

Focus-animal observations by Hofmann et al. (2025) documented the times at which these chicks from both hatching methods first consumed feed and water. OH chicks showed that, on average, they took their first feed approx. 10 h after hatching and drank from the nipple drinkers 16 h after hatching. The HH chicks consumed feed for the first time 28 h after hatching, i.e., immediately upon arrival in the house, and drank from the nipple drinkers on average 29 h after hatching.

De Jong et al. (2019) reported comparable findings in a study conducted on commercial farms using Ross 308 chicks, where on-farm hatched (OH) chicks were significantly heavier at day 1 (approx. 5.4 g) and day 7 (approx. 11.5 g) compared to the control group sourced from the hatchery.

Chick quality, evaluated using the Pasgar^ࣩ^ Score (reflex, navel, legs, beak, and belly), did not differ significantly in this study. This outcome may be attributed to the veterinarians or experienced staff selecting non-viable on-farm-hatched chicks, rather than farm employees in other studies. This approach also resulted in similar mortality rates between the hatching groups after 7 days and at the end of the 42-day fattening period. In contrast, Souza da Silva et al. (2021) conducted an experiment with 1,155 chicks per group (hatchery-hatched, on-farm hatched, and early feeding chicks) and observed significantly poorer chick quality in OH chicks compared to control groups. This was assessed by righting test, chick length, body weight (including residual yolk) and scoring for navel, beak, and hock quality. In that study there were higher mortality rates in the OH group compared to control groups (Souza da Silva et al., 2021), too. This is consistent with findings by de Jong et al. (2019, 2020). These differences may be explained by the greater difficulty in on-farm selection than that in the hatchery assembly line. The level of experience is also a critical factor in this context.

### Behavioral tests

While no significant differences in the number of animals entering the radius were observed between HAT and NOT, the OH group consistently had a higher number, especially at DL 1.

At DL 7, a marginally higher number of OH chicks gathered near the human; however, the latency between OH and HH groups remained equivalent. Across all 7 PP, no OH chicks made contact with the human, whereas HH chicks required nearly the entire 180-second observation period and touched the human infrequently (only 2 of 112 observations).

OH chicks might already be familiar with their hatching environment. In contrast, HH chicks must adapt to new auditory, visual, olfactory, and social stimuli. The increased motivation to search for feed, resulting from a longer fasting period after hatching, particularly at DL 1, may account for HH chicks' greater focus on eating and drinking rather than on interacting with unfamiliar objects or humans in the poultry house. This is also shown by louder vocalization of HH chicks at DL 1 (Hofmann et al., 2025). Video analyses demonstrated that the proportion of OH chicks displaying specific behavior patterns (such as drinking, eating, resting, and walking) remained stable. Although HH chicks initially concentrated on eating, drinking, and walking after approximately 24 hours, the proportion of individuals resting became comparable to that observed in OH chicks (not published).

Another possible explanation for the behavior of OH chicks toward humans could be their habituation to human presence, which results from intensive care during hatching in the barn. This care included 4 control rounds per day, each lasting at least 30 minutes, rigorous selection of non-viable chicks, and eggshell cleaning, all of which involved frequent movement through the stable. Stocker et al. (2025) reported comparable effects, as multiple rounds of gentling programs accustoming groups of approximately 220 Ranger Classic broilers to humans resulted in increased interaction with humans in various behavioral tests compared to the control group.

However, Stocker et al. (2025) findings suggest that habituation to humans does not necessarily reduce fearfulness, as no differences were observed between gentling and control groups in the Novel Object Test.

In this study the OH chicks did not exhibit avoidance behavior toward humans, whereas the HH chicks, encountering humans in the barn for the first time, took longer to approach them. Jones (1995, 1993) suggested that mere presence fosters familiarity through repeated exposure to a particular environment or to consistently present companions, which may also result in habituation to humans who are consistently present but neither reward nor punish the animals.

For the NOT, by the end of the first week of life (DL 7), HH chicks entered the radius around the unknown object more frequently and with shorter latency, and touched it more rapidly than OH chicks. One possible explanation may be that OH chicks, feeling more secure and less anxious in their familiar environment, approached unfamiliar objects more quickly at DL 1. In contrast, HH chicks required time to acclimatize to their new environment and gradually reduced their fear of novel stimuli with age (Jessen et al., 2021).

Conducting behavioral observations at later life stages and on farms with different management procedures could provide additional insight.

This would help determine if the hatching method has a lasting effect on chickens. Previous research by Hedlund et al. (2019) found that hatchery-hatched layers tend to exhibit more agonistic behavior toward other hens, including feather pecking and comb damage and showed fear related behavior.

The effect of age could not be confirmed in this study. Giersberg et al. (2021) found that animals may become less fearful as they mature, with a higher number of chicks at older ages in small groups of 1,150 per pen. In contrast, this study observed fewer animals at DL 7 than at DL 1, and the latency period was longer.

It is possible that animals in smaller groups become accustomed to humans more rapidly.

### Urofecal corticosterone metabolites

To measure stress hormone concentrations in chicks using a non-invasive method that avoids the stress effects of blood sampling (Romero et al., 2005), urofecal samples were collected. For this, the validated method described by Wolf et al. (2024) was used, which showed that an important parameter to consider when collecting urofecal samples from chickens to determine ufGCM is that the samples should be frozen within 4 hours after defecation. The time of day at which the samples are collected does not significantly affect the results (Wolf et al., 2024), and it is also possible to collect droppings and pool them. This was important for this study because the chicks' droppings in the first days of life are very small, and to proceed with the immunoassay, an approx. amount of 0.05 g dry-weight is needed.

It is noticeable that the ufGCM concentrations at the day of hatching (DL 0) are more than 4 times higher compared to 5 days after hatching for both hatching forms. The high concentration of corticosterone metabolites found on the day of hatching is most likely due to an increased release of glucocorticoids at the end of incubation (Wentworth et al., 1985). Glucocorticoids effectively increase plasma triiodothyronine (T3**)** concentration by reducing hepatic T3-degrading activity. The interaction between the thyroid and adrenal axes also extends to the hypothalamo-pituitary axis, as the corticotropin-releasing hormone elevates glucocorticoids (de Groef et al., 2013). Glucocorticoids and thyroid hormones are involved in the preparation for hatching, namely by increasing blood flow to the lungs and by promoting the development of the surfactant system in the embryonic lung of the chicken (Blacker et al., 2004). The hatching climax, characterized by great activity, is a chick's escape from the shell at an appropriate stage of development. The chick performs many coordinated movements. The process of pipping and hatching is a drastic change and can be regarded as stressor (Decuypere et al., 2006). It took approximately 4 days for the elevated levels stress hormone concentrations (Mean: approx. 1,700 ng/g) in chicks to return to stable levels (Mean: approx. 400 ng/g).

Similar to what was hypothesized and also described by Hedlund et al. (2019), namely that there are differences in behavior (feather pecking, comb damage, and weight differences) as well as the increased blood corticoid values in laying hens due to handling in the hatchery, the ufGCM concentrations (P = 0.057) between the 2 hatching forms tended to be different. Statstically significant differences might have been demonstrated by a larger sample size, i.e. if for example more urofecal samples were collected within a single fattening run or across more than 2 production periods. Consequently, further calculations, including those involving the individual PPs and the hatching form, could not be performed.

It is therefore assumed that the stress level, or rather the increase of glucocorticoids in the chicks during hatching is already so high that the sorting and other procedures at the hatchery did not have an additional major impact.

However, within the constraints of this study design, HH chicks exhibited a marginally higher concentration of ufGCM compared to OH chicks.

What might result from the fasting phase after hatching. Wolf et al. (2024) showed that a 12-hour fasting period before loading for slaughter results in an approximately 3-fold increase of ufGCM concentrations. This is explained by the fact that today's broiler breeding lines, even the slow-growing ones, are bred to reach a higher weight in a shorter time through increased appetite and metabolism. The fasting procedure, thus, likely puts stronger physiological pressure on these high-metabolizing animals. The chicks hatched in the barn have access to feed and water immediately after hatching. Therefore, they do not undergo a fasting phase during transportation from the hatchery to the farm.

## Conclusion

In summary, this field study conducted on a single farm indicates that the hatching method, particularly the initial hours post-hatching, may influence chicken behavior and ufGCM concentrations. The findings suggest that early exposure to humans after hatching could foster a more familiar relationship between chickens and humans. To the authors' knowledge, this is the first study to measure stress levels in broiler chickens from different hatching methods using ufGCM. While no statistically significant differences were detected between hatching methods, a trend toward higher ufGCM concentrations was observed in HH chicks, potentially reflecting the fasting period from hatching to barn arrival.

Verification of this trend requires additional field studies on other farms, utilizing larger sample sizes and a greater number of PP than those included in the present study.

Further investigations, such as noise-level measurements, individual-animal observations, and activity-pattern recordings, could provide additional insights and expand our understanding of how hatching methods affect broiler chicken welfare.

## CRediT authorship contribution statement

**Sabine Christa Margarete Vossler:** Writing – original draft, Investigation, Formal analysis, Data curation. **Anna Schwarz:** Project administration. **Stephanie Schäfers:** Formal analysis. **Chadi Touma:** Methodology, Formal analysis, Conceptualization. **Tanja Wolf:** Methodology, Formal analysis, Conceptualization. **Nicole Kemper:** Supervision. **Birgit Spindler:** Writing – review & editing, Methodology, Conceptualization.

## Disclosures

The authors declare that the research was conducted in the absence of any commercial or financial relationships that could be construed as a potential conflict of interest.

## Appendix Supplementary materials

Image, application 1

## Footnotes

Supplementary material associated with this article can be found, in the online version, at doi:10.1016/j.psj.2026.107358.
