# Supplementary material for: Hatching on-farm under field conditions –a comparison of fear-related response and urofecal glucocorticoid metabolite levels in broiler chicks hatched on-farm and in the hatchery
Source: Poult Sci. 2026 Jun 27;105(11):107358. doi: 10.1016/j.psj.2026.107358 (PMC13449652; doi:10.1016/j.psj.2026.107358)
Supplement: Supplementary file 1 [file mmc1.docx]

| **Production period** | **Hatching**  **form** | **Tempratur (**°C)  **and air humidity (rel%)** | | | | | |
| --- | --- | --- | --- | --- | --- | --- | --- |
|  |  | **DL1** | **DL2** | **DL4** | **DL5** | **DL7** | **DL42** |
| **Recommendation** |  | 30°C  70% | 30°C  70% | 28°C  65% | 28°C  50% | 27°C  50% | 20°C  50% |
| **PP1** | OH | 33.1°C  61% | 32.7°C  60% | 31.5°C  61% | 30.8°C  61% | 29°C  62% | 21.9°C  82% |
|  | HH | 33.1°C  61% | 32.7°C  60% | 31.5°C  60% | 30.8°C  61% | 29°C  62% | 21.9°C  82% |
| **PP2** | OH | 33°C  60% | 32.4°C  69% | 31.2°C  62% | 30.4°C  61% | 29.3°C  67% | 20.4°C  81% |
|  | HH | 33°C  56% | 32.4°C  64% | 31.7°C  58% | 30.5°C  57% | 29.4°C  60% | 20.4°C  72% |
| **PP3** | OH | 32.9°C  48% | 32.4°C  50% | 31.7°C  46% | 30.5°C  52% | 29.3°C  53% | 20.7°C  73% |
|  | HH | 33°C  57% | 32.4°C  61% | 31.1°C  58% | 30.5°C  60% | 29.3°C  61% | 21.1°C  83% |
| **PP4** | OH | 32.9°C  46% | 32.3°C  48% | 31°C  51% | 30.3°C  54% | 29.2°C  57% | 20.3°C  81% |
|  | HH | 30.9°C  47% | n.a.  n.a. | 30.9°C  47% | 29.7°C  48% | 29.3°C  52% | 20.2°C  71% |
| **PP5** | OH | 33°C  48% | 32.5°C  48% | 31.2°C  49% | 30.4°C  51% | 29.3°C  55% | 19°C  72% |
|  | HH | 30.5°C  59% | 31.2°C  57% | 29.9°C  60% | 29.3°C  65% | 28.2°C  69% | 21.1°C  76% |
| **PP6** | OH | 32.7°C  59% | 32°C  68% | 30.8°C  74% | 30.2°C  74% | 29°C  75% | 25.1°C  88% |
|  | HH | 32.7°C  55% | 32.1°C  60% | 30.8°C  65% | 30.3°C  64% | 29.1°C  65% | 24.7°C  81% |
| **PP7** | OH | 33.2°C  63% | 32.6°C  64% | 31.4°C  62% | 30.6°C  65% | 29.5°C  65% | 23.3°C  79% |
|  | HH | 33.1°C  71% | 32.7°C  73% | 31.4°C  71% | 30.7°C  74% | 29.5°C  73% | 23°C  88% |

Table A: Temperatur (C°) and air humidity (rel%) of each hatching group (OH = on-farm hatched; HH = hatchery-hatched) from the seven production periods (PP1-7) of broiler chicken (Ranger classic) during the first week of life (Day of life (DL) 1, 2, 4, 5, 7) and at the end of fattening (DL42) (Recommendation from Aviagen Ross Broiler Management Handbook 2025

Table B: Descriptive Statistics of Number of chicks per Test and Day of Life using Microsoft Excel (version 2016; Microsoft Corporation, edmond,WA)

| **Day of Life** | **Hatch-form** | **Test** | **Min** | **Max** | **Mean Value** | **Median** | **SD** |
| --- | --- | --- | --- | --- | --- | --- | --- |
| 1 | OH | HAT | 8 | 142 | 64,14 | 46 | 52,40 |
|  | HH | HAT | 5 | 125 | 38,86 | 18 | 48,29 |
|  | OH | NOT | 80 | 187 | 128,86 | 134 | 33,68 |
|  | HH | NOT | 17 | 176 | 70,71 | 40 | 66,82 |
| 7 | OH | HAT | 0 | 150 | 47,57 | 27 | 61,37 |
|  | HH | HAT | 0 | 142 | 32,86 | 6 | 53,64 |
|  | OH | NOT | 24 | 308 | 106,71 | 61 | 99,73 |
|  | HH | NOT | 8 | 244 | 123,00 | 131 | 79,90 |

Table C: Descriptiv Statistics Latency in seconds of Novel Object Test per Hatch-form and Day of Life for Radius 25cm and Touch using Microsoft Excel (version 2016; Microsoft Corporation, Edmond,WA)

| Day of Life | Hatch-form | Latency NOT | Min | Max | Mean Value | Median | SD |
| --- | --- | --- | --- | --- | --- | --- | --- |
| 1 | OH | Radius 25cm | 1 | 180 | 37,84 | 29 | 39,51 |
|  | HH | Radius 25cm | 2 | 180 | 89,82 | 85 | 74,21 |
|  | OH | Touch | 1 | 180 | 72,86 | 56 | 57,76 |
|  | HH | Touch | 5 | 180 | 136,27 | 180 | 65,52 |
| 7 | OH | Radius 25cm | 1 | 180 | 69,75 | 54 | 64,01 |
|  | HH | Radius 25cm | 1 | 180 | 61,18 | 33 | 60,61 |
|  | OH | Touch | 39 | 180 | 151,77 | 180 | 45,00 |
|  | HH | Touch | 13 | 180 | 141,42 | 180 | 55,78 |
|  |  |  |  |  |  |  |  |

Tabel D: Statistics ufCORT (ng/ g) P = 0.057

| **Day of life** | **Hatch-form** | **Minimum** | **Maximum** | **Median** | **Mean Value** | **Standard deviation** |
| --- | --- | --- | --- | --- | --- | --- |
| 0 | OH | 777.02 | 2224.21 | 1612.21 | 1562.73 | 439.63 |
| 0 | HH | 1079.88 | 2599.76 | 1655.66 | 1730.79 | 399.84 |
| 1 | OH | 674.56 | 1465.43 | 965.88 | 970.49 | 173.07 |
| 1 | HH | 610.83 | 1860.44 | 983.10 | 1035.16 | 313.79 |
| 2 | OH | 458.52 | 988.56 | 701.19 | 684.37 | 140.58 |
| 2 | HH | 431.13 | 1165.47 | 665.04 | 695.00 | 164.55 |
| 4 | OH | 374.90 | 504.18 | 423.04 | 434.58 | 56.00 |
| 4 | HH | 315.39 | 524.74 | 438.57 | 423.46 | 87.73 |
| 5 | OH | 366.97 | 688.40 | 440.17 | 467.04 | 119.83 |
| 5 | HH | 349.76 | 440.31 | 397.43 | 396.33 | 28.83 |
| **overall** | **OH** | **366.97** | **2224.21** | **895.44** | **1006.74** | **491.36** |
| **overall** | **HH** | **315.39** | **2599.76** | **880.91** | **1019.82** | **543.27** |
